# Intravesical Botulinum Toxin Injection Plus Hydrodistention Is More Effective in Patients with Bladder Pain-Predominant Interstitial Cystitis/Bladder Pain Syndrome

**DOI:** 10.3390/toxins16020074

**Published:** 2024-02-01

**Authors:** Wan-Ru Yu, Jia-Fong Jhang, Hann-Chorng Kuo

**Affiliations:** 1 Department of Nursing, Hualien Tzu Chi Hospital, Buddhist Tzu Chi Medical Foundation, Hualien 97002, Taiwan; wry@tzuchi.com.tw; 2 Institute of Medical Sciences, Tzu Chi University, Hualien 97004, Taiwan; 3 Department of Urology, Hualien Tzu Chi Hospital, Buddhist Tzu Chi Medical Foundation, Hualien 97002, Taiwan; alur1984@hotmail.com; 4 Department of Urology, School of Medicine, Tzu Chi University, Hualien 97004, Taiwan

**Keywords:** botulinum toxin (BoNT-A), bladder pain, interstitial cystitis/bladder pain syndrome (IC/BPS)

## Abstract

Intravesical botulinum toxin A (BoNT-A) injections are included in the interstitial cystitis/bladder pain syndrome (IC/BPS) treatment guidelines. However, the IC phenotype suitable for treatment with BoNT-A has not been clarified. Therefore, we identified the factors influencing treatment outcomes for intravesical BoNT-A injections in patients with non-Hunner IC/BPS (NHIC). This retrospective study included patients with NHIC who underwent 100 U BoNT-A intravesical injections over the past two decades. Six months after treatment, treatment outcomes were assessed using the Global Response Assessment (GRA). Outcome endpoints included GRA, clinical symptoms, urodynamic parameters, urine biomarkers, and the identification of factors contributing to satisfactory treatment outcomes. The study included 220 patients with NHIC (42 men, 178 women). The satisfactory group (*n* = 96, 44%) had significantly higher pain severity scores and IC symptoms index, larger maximum bladder capacity (MBC), and lower 8-isoprostane levels at baseline. Logistic regression revealed that larger MBC (≥760 mL) and bladder pain predominance were associated with satisfactory outcomes after BoNT-A injection. Subjective parameters and pain severity scores improved significantly in patients with bladder pain-predominant IC/BPS after BoNT-A injection. Thus, NHIC patients with bladder or pelvic pain are more likely to experience satisfactory outcomes following intravesical BoNT-A injections.

## 1. Introduction

Interstitial cystitis/bladder pain syndrome (IC/BPS) is a complicated and puzzling health condition. Patients with IC/BPS experience pain in the bladder or pelvic cavity and lower urinary tract symptoms, including frequent urination, urgency, and nocturia [1]. Although IC/BPS is not lethal, the pain and urinary symptoms significantly impact the patient’s quality of life and mental health. The underlying pathophysiology of this condition is not fully understood, and effective treatments with long-lasting outcomes are lacking [2]. Furthermore, the diagnostic criteria vary widely, and the IC/BPS prevalence estimates range from 0.045% to 6.5% in women and 0.008% to 4.2% in men [3].

Although the pathophysiology of IC/BPS is not well understood, the clinical and pathological presentation of patients with Hunner lesions (“IC” or “HIC”) and patients without Hunner lesions (“BPS” or non-Hunner IC “NHIC”) differ [4]. Chronic inflammation induced by autoimmunity, infection, external substances, or unidentified reasons causes HIC, whereas neurogenic inflammation, neurogenic hyperactivity, or defects of the urothelial barrier cause NHIC [5]. In addition, patients with IC/BPS often have functional somatic disorders, including irritable bowel syndrome, fibromyalgia, chronic fatigue syndrome, panic attacks, depressive disorders, migraines, and vulvodynia [6]. Regardless of the phenotype, pain is the main symptom of IC/BPS, and pain relief, behavioral modification, and stress reduction should be provided as a multifaceted, multidisciplinary approach to treating IC/BPS [2]. IC/BPS involves whole-body somatic symptoms [7], which result in chronic bladder nociceptive signals due to central sensitization and heightened neuronal responsiveness [8].

Botulinum toxin (BoNT-A) has been widely used to improve several human symptoms, such as neuropathic pain or any pain reduction, migraine, excessive muscle stiffness, spasticity, dystonia, stroke, and sclerosis, autonomic dysfunction, and even disease in children [9]. It has been approved for the treatment of neurogenic detrusor overactivity and idiopathic overactive bladder (OAB) refractory to conventional medical therapy [10]. It is a neurotoxin protein with paralytic effects. In IC/BPS, urothelial deficiency is commonly considered a leading factor. The functional or quantitative impairment of the urothelial barrier allows direct contact of irritating urinary subnoxious with the suburothelial layer, triggering inflammatory responses or afferent nerve stimulation [11]. The noxious stimulation of sensory nerve endings prompts the release of various chemicals, activating specific pain receptors. Additionally, the local tissue inflammation induced by accumulating chemicals such as histamine, bradykinin, and substance P contributes to further nerve-ending stimulation, leading to peripheral sensitization [9]. BoNT-A rapidly and strongly binds to presynaptic cholinergic nerve terminals and cleaves the synaptosomal-associated protein 25 kDa (SNAP-25). Cleavage of SNAP-25 inhibits exocytosis of acetylcholine and other neuropeptides from nerve terminals [12], causing paralysis of the affected neuromuscular junctions [13]. BoNT-A, which elicits anti-inflammatory and antinociceptive effects, significantly attenuates pain and decreases the frequency of urination in patients with refractory IC/BPS [14]. Therefore, intravesical BoNT-A injection has been incorporated into the IC/BPS treatment guidelines [15]. However, the most suitable phenotype of patients with IC/BPS for intravesical BoNT-A injection treatment has not been identified.

The purpose of this study was to determine the real-life factors associated with satisfactory treatment outcomes after intravesical BoNT-A injections in patients with IC/BPS.

## 2. Results

### 2.1. The Subjective and Objective Baseline Characteristics of Patients with NHIC

From January 2000 to January 2023, 220 NHIC patients (42 males, 178 females) were enrolled in the study. The mean age was 53.6 ± 13.2 years, and the mean disease duration was 13.5 ± 10.0 years. Ninety-six patients (43%) reported satisfactory outcomes (Global Response Assessment, GRA ≥ 2), whereas 124 (56%) patients reported unsatisfactory treatment outcomes (GRA ≤ 2). Patients with a satisfactory outcome had significantly higher interstitial cystitis symptom index (ICSI) (13.1 ± 3.76 vs. 12.1 ± 3.67, *p* = 0.043), interstitial cystitis problem index (ICPI) (12.7 ± 3.37 vs. 11.3 ± 3.17, *p* = 0.002), and bladder or pelvic pain severity on the numerical rating pain scale (NRS, 5.44 ± 2.47 vs. 4.55 ± 2.73, *p* = 0.004) compared with the corresponding parameters in the unsatisfactory group. A total of 57% of patients with the bladder pain-predominant phenotype (NRS ≥ 5) was noted in all patients. The percentage of patients with the bladder pain-predominant subtype was significantly higher in the satisfactory group compared with the percentage in the unsatisfactory group (70.8% vs. 46.8%, *p* < 0.001). There are no significant differences in age, gender, IC duration, or comorbidities with autonomic nervous systems (ANS) diseases such as sleep disorders, depression, anxiety, hypertension, asthma, irritable bowel disease, arrhythmia, heart disease, recurrent urinary tract infection, gastroesophageal reflux disease, myofascial pain syndrome, or another immune disease between satisfactory and unsatisfactory groups (Table 1).

In addition, we compared the objective data of videoreodynamic study (VUDS) and uroflowmetry parameters, potassium chloride (KCl) test, and maximum bladder capacity (MBC) and glomerulation grade after cystoscopic hydrodistention. There are no significant differences in VUDS and uroflowmetry parameters, including the first sensation of filling, full sensation, cystometric bladder capacity, detrusor pressure, maximum flow rate, voided volume, postvoid residual, KCl test, and glomerulation grade between groups at baseline. However, the maximum bladder capacity (MBC) after cystoscopic hydrodistention was higher in patients with satisfactory treatment outcomes compared with unsatisfactory treatment outcomes (705 ± 184.3 vs. 647.5 ± 189.2 mL, *p* = 0.025) (Table 2).

### 2.2. The Baseline Urine Biomarkers of Patients with NHIC

Further, Table 3 shows the urinary biomarker concentrations in each treatment outcome group. The 8-isoprostane levels, indicating oxidative stress, were lower in the urine of patients in the satisfactory group compared with the levels in the unsatisfactory group (45.9 ± 46.5 vs. 120.5 ± 260.0, *p* = 0.022) at baseline. No other significant difference was detected in inflammatory biomarkers and oxidative stress urine biomarkers between the satisfactory or unsatisfactory groups, including IL-8, CXCL10, MCP-1, BDNF, eotaxin, IL-6, MIP-1β, RANTES, PGE2, TNF-α, 8-OHDG, and TAC. However, when we compared with the control group who received the suburethral sling procedure, there were significant differences among the groups of NHIC patients and the control group in urine biomarkers including MCP-1, NGF, eotaxin, IL-2, PGE2, 8-OHDG, and 8-isoprostane.

### 2.3. The Changes of Subjective and Objective Characteristics of Patients with NHIC after Intravesical Botulinum Toxin A Injection Treatment

Further comparison of the parameters of intravesical BoNT-A injection treatment outcome between unsatisfactory and satisfactory groups in the patients with NHIC showed significant improvement in the changes of subjective parameters including IC symptoms and problem index, and pain severity (ΔICSI: −3.12 ± 4.38 vs. −5.46 ± 4.84, *p* = 0.001; ΔICPI: −3.32 ± 4.57 vs. −6.83 ± 5.06, *p* = <0.001; ΔNRS: −1.09 ± 2.98 vs. −2.89 ± 3.21, *p* = <0.001) in the satisfactory group compared with the unsatisfactory group. However, the changes in uroflowmetry variables were not significantly different between groups (Table 4).

**Figure 1:**
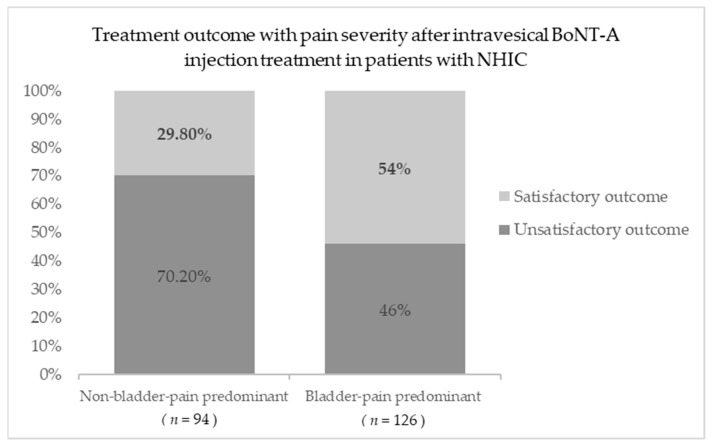
The rate of satisfactory s to BoNT-A treatment between NHIC patients with baseline pain score NRS ≥ 5 and NRS < 5. BoNT-A, botulinum toxin A; NHIC, non-Hunner IC/BPS.

Moreover, this study further divided into two groups, non-bladder pain-predominant phenotype or bladder pain-predominant phenotype with NHIC patients based on NRS, to explore the correlation between treatment outcome after intravesical BoTN-A injection and pain severity; NHIC with a higher grade of bladder or pelvic pain at baseline exhibited a significantly higher rate of satisfactory treatment outcomes after intravesical BoNT-A injection treatment. In the bladder pain-predominant group, 54% (68/126) of patients achieved satisfactory treatment outcomes, while only 29.8% (29/94) of patients in the non-bladder pain-predominant group achieved satisfactory treatment outcomes (*p* < 0.001) (Figure 1).

### 2.4. An Analysis of the Factors Associated with Satisfactory Outcomes after Intravesical BoNT-A Injection Treatment in Patients with NHIC

**Figure 2:**
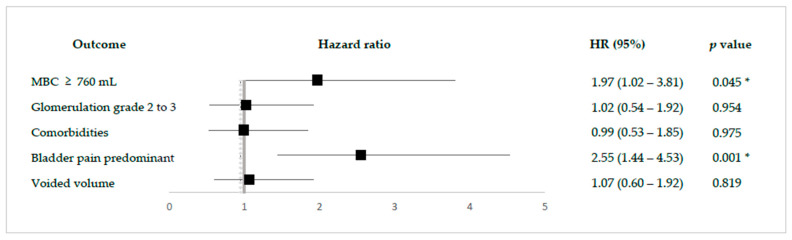
The forest plot results from the logistic regression for predicting satisfactory treatment outcome (GRA ≥ 2) effect factors after intravesical BoNT-A injection. * Significant *p* < 0.05.

Lastly, a multivariate logistic regression analysis was performed using the parameters correlated with treatment outcomes to tap into the satisfactory treatment outcome population who received the suitable intravesical BoNT-A injection treatment. Based on clinical characteristics, possible predictors would be analyzed, including bladder capacity and glomerulation grade under anesthesia of bladder condition, comorbidities to examine other associated factors such as autonomic nervous system disease, and phenotypes that lead to a satisfactory treatment outcome after the intravesical BoNT-A injection, like bladder-pain predominant or lower urinary tract symptoms predominant; significant predictors are larger MBC (≥760 mL) (Hazard ratio: 1.97, 95% CI: 1.02–3.81, *p* = 0.045) and as a bladder pain-predominant phenotype, NHIC patients (Hazard ratio: 2.55, 95% CI: 1.44–4.53, *p* = 0.001) at baseline would more likely achieve a satisfactory treatment outcome after an intravesical BoNT-A injection (Figure 2).

Nevertheless, several unfavorable adverse events were observed following a BoNT-A injection, including hematuria in 6 (2.7%) patients, urinary tract infections in 4 (1.8%) patients, and mild dysuria in 36 (16%) patients. However, no patients reported acute or chronic urinary retention.

## 3. Discussion

This large cohort study aimed to determine the relationship between the intravesical BoNT-A injection treatment outcome and NHIC clinical phenotypes. A satisfactory outcome was reported by 43% of NHIC patients after an intravesical BoNT-A injection, and 70.8% of patients had bladder or pelvic pain as the predominant phenotype. This retrospective analysis aims at identifying real-world patients who are more suitable for an intravesical BoNT-A injection by *t*-test and multinomial logistic regression to compare different outcome groups and try to find some significant parameters and further estimate subjective and objective factors in an attempt to predict the factors that influence the expected treatment outcomes that try to build a scoring system. However, the results of this study revealed that the patient-reported ICSI, ICPI, and NRS pain scores correlated with treatment outcomes and that satisfactory BoNT-A treatment outcomes would be associated with larger bladder capacity and pain-predominant phenotype NHIC patients. In the previous study, intravesical injections of BoNT-A into the bladder have been proven effective in reducing the inflammatory processes within the bladder wall associated with the pathogenesis of IC/BPS. This effect leads to the decreased expressions of sensory and inflammatory receptors, ultimately alleviating bladder pain and concurrently increasing MBC during cystoscopic hydrodistention. Notably, although hydrodistention may transiently enhance bladder capacity and pain severity, the therapeutic mechanisms of BoNT-A and hydrodistention differ [16,17]. Thus, factors contributing to satisfactory BoNT-A treatment outcomes are related to the predominant bladder pain phenotype.

First, this study reveals that NHIC is most commonly seen in middle age [3], and the female-to-male ratio is 8:2. Additionally, NHIC is often a non-organic disease characterized by a normal appearance with an internal variation of chronic inflammation and pain. torturing with disruptive bladder pain and lower urinary tract symptoms in patients with IC/BPS. Depending on the type of pathology, pearlized with or without Hunner lesion, an immunological inflammatory response often coexists with cell clonal expansion and epithelial denudation in HIC, and functional defects in the urothelial barrier, neurogenic inflammation, neural hyperactivity, and extravesical disorders in NHIC [5]. In this study, in patients who received the intravesical BoNT-A injection, even the symptom severity and pain scores of patients with the satisfactory outcome group were higher at baseline, but the change in symptom scores was also more remarkable for the satisfactory group compared to the unsatisfactory group.

Therefore, patients with NHIC usually have functional somatic syndromes, central sensitization, and inter-organ crosstalk [18]. Sensory input from the diseased bladder to the spinal cord may also result in a convergent sensation in the central nervous system, leading to central sensitization (crosstalk) of visceral or musculoskeletal organs and hypersensitivity symptoms [19,20]. Inflammation, pain, and the psychological state might activate the sympathetic and ANS and hypothalamic pituitary adrenal axis [21,22,23]. Infection, injury, or stress [24] can activate immune cells to release inflammatory mediators and promote healing via phagocytosis of foreign bodies or tissue debris. If the inflammation does not resolve, the immune system is repeatedly triggered, resulting in chronic inflammation [25].

In this study, NHIC patients have significantly higher urine inflammation cytokines and oxidative stress biomarkers like MCP-1, NGF, eotaxin, IL-2, PGE2, 8-OHdG, and 8-isoprostane compared with the control group; a more substantial implication suggests that chronic inflammation in patients with IC/BPS is persistently ongoing in the bladder. Notably, significantly higher sensory receptors (TRPV1, TRPV4, and sigma-1 receptors), inflammatory proteins, and pro-apoptotic proteins were noted in the bladders of IC/BPS patients [26]. Additionally, TRPV4 and sigma-1 receptors would transmit neuropathic pain from peripheral nerve injury to the spinal cord [27,28,29]. Thus, BoNT-A could cleave SNAP25, decrease cell-surface expression of TRP channels (TRPV1, TRPA1), internalized BoNT-A proteins paralyze neurons by reducing acetylcholine exocytosis [12,30], inhibit sensory neurotransmitter release from isolated bladder preparations in rats [31] and impede degranulation in both human and murine mast cells [32,33], and inhibit the secretion of inflammatory mediators such as histamine and TNF-α, further inhibiting the inflammatory process and antinociceptive effects [14,34].

Therefore, patients with unsatisfactory treatment outcomes had higher baseline levels of urinary 8-isoprostane, a biomarker of oxidative stress that might indicate a severe inflammation status in the unsatisfactory group. Thus, BoNT-A injection as an effective treatment for IC/BPS has been included in the American Urological Society (AUA) and Japanese Urological Association (JUA) guidelines; a repeated injection is required [2,5]. Other bladder treatments to improve blood supply and eradicate inflammation, such as platelet-rich plasma (PRP) or low-energy shock wave (LESW) therapy, may elicit a more satisfactory outcome in these patients [35].

In addition, this study also noted more ANS comorbidities in all patients with NHIC, signifying that NHIC is not a single disorder but rather encompasses a spectrum of distinct disorders [36]. Chronic inflammation and oxidative stress damage the bladder urothelium [37], and stress increases the voiding frequency, somatic sensitivity, urinary bladder nerve growth factor, and BDNF expression [38]. Based on these therapeutic mechanisms, reducing inflammation, pain control, protecting epithelial denudation, and stabilizing psychological status are the treatment goals.

Due to unclear etiology, this retrospective analysis aims to identify patients who are more suitable for intravesical BoNT-A injection and tries to find some significant parameters. Importantly, NHIC demonstrates a varied and complex nature, contributing to the heterogeneity observed in its pathophysiological manifestations. Urothelial deficits, impaired urothelial cell differentiation, chronic inflammation, and central nervous system sensitization may contribute to IC symptoms and morphological changes in the bladder wall. In this study, patients with satisfactory responses to BoNT-A treatment had higher bladder pain scores and MBC. However, the two groups had similar bladder glomerulation after cystoscopic hydrodistention, suggesting that the patients responsive to BoNT-A had more neuronal hyperplasia than bladder wall inflammation and fibrosis. In other words, NHIC patients possibly had more uncertain and various etiology leading to inflammatory, somatic symptoms and psychological stress resulting in secondary phenomena in the bladder [39,40].

Currently, no effective and durable treatments for NHIC are available; the most suggested treatments include conservative treatments of behavior modification, medical treatments of pain control and anti-inflammation, intravesical instillation or bladder wall injection of DMSO or hyaluronic acid instillation, and novel treatments such as electrostimulation [5] or PRP and LESW. Most guidelines list treatment options for NHIC, but treatment priority has not been established. Recent guidelines also suggest personalized treatments based on phenotype rather than step-by-step treatments [2]. Our results support this personalized approach to treatment; NHIC patients with a pain-predominant phenotype are more responsive to an intravesical BoNT-A injection. Repeat BoNT-A injections may result in a durable satisfactory outcome. Additionally, in treating lower urinary symptoms in patients with OAB, not only is the concentration administered through oral intake lower than that drug instilled intravesically, but intravesical instillation of anti-muscarinic treatment can reduce systemic adverse events in elderly patients [41]. When we point to the intravesical BoNT-A with bladder volume, BoNT-A’s impact on muscle paralytic function and its ability to alleviate bladder spasms by reducing involuntary contractions and enhancing overall bladder volume might positively influence voiding volume in individuals suffering from IC/BPS. The rate of patients who developed urinary retention after BoNT-A injection is lower than that which occurred in patients with an overactive bladder. We have observed that OAB women had higher PVR volume and lower voiding efficiency than those in IC/BPS after BoNT-A injections, further indicating that an intravesical BoNT-A injection is safe in patients with IC/BPS [42]. It is hypothesized that the detrusor contractility might be more susceptible to BoNT-A in OAB due to muscle paralysis. Still, it has a more anti-inflammatory effect with less detrusor contractility effect in IC/BPS. Some patients with IC/BPS will have adverse events of difficulty in urination due to a decrease in bladder fullness or urge sensation, but the contractility is usually not affected after the BoNT-A injection. BoNT-A may have different therapeutic targets on OAB and IC/BPS. However, further investigation is needed to clarify the true mechanism of this difference, and it is crucial to underscore that a comprehensive and intensive precision multimodal therapeutic approach is indispensable for the effective management of IC/BPS. This study has revealed that NHIC patients as a pain-predominant phenotype would benefit from an intravesical BoNT-A injection.

There are several limitations to this study. First, a retrospective analysis of patient data from a single center was conducted. Second, even in trials with the same inclusion and exclusion criteria, selection bias for NHIC patients may exist. Third, rigorous studies using different phenotype classifications should be conducted, including patients with different MBC and glomerulation grades after cystoscopic hydrodistention. Lastly, this study reported the association between the pain phenotype and self-reported treatment outcomes after an intravesical BoNT-A injection; however, external validity is needed. Therefore, a multi-center, prospective study should be conducted.

## 4. Conclusions

NHIC patients with a bladder or pelvic pain-predominant phenotype are more likely to experience a satisfactory treatment outcome following intravesical BoNT-A injections.

## 5. Materials and Methods

### 5.1. Patients

This prospective study enrolled 220 patients with IC/BPS and NHIC under the cystoscopic hydrodistion diagnostic who had received, for the first time, an intravesical BoNT-A 100-unit injection. All patients were diagnosed with IC/BPS based on characteristic symptoms, glomerulations, and petechial and mucosal fissures after cystoscopic hydrodistention under anesthesia [43]. The patients were included in several previous studies (https://clinicaltrials.gov, accessed on 17 December 2023, NCT01969773 and NCT03076762). All patients received at least two treatment modalities, including lifestyle and behavior modification, antinociceptive medication treatment, cystoscopic hydrodistention, and intravesical hyaluronic acid instillation [2]. Some patients were treated with repeated BoNT-A injections; however, only the outcome of the first BoNT-A injection was recorded in this study. All patients were thoroughly screened and were not enrolled if they failed to meet the inclusion criteria of the European Society for the Study of IC/BPS [44]. Data from previous clinical trials of BoNT-A injections for patients with NHIC were retrospectively analyzed. IC questionnaires were collected, including ICSI, ICPI, and NRS, at baseline and after treatment, with GRA as the primary endpoint. Additionally, a uroflowmetry study was conducted to measure maximum flow rate, voided volume, and post-residual urine before and after treatment. Additionally, the VUDS examination, urine specimen collection, and cystoscopic hydrodistention were performed before an intravesical BoNT-A injection.

### 5.2. Assessments Questionnaires and Examination

The effectiveness of an intravesical BoNT-A injection was assessed six months after the injection relative to the baseline using the GRA. The GRA consists of a 7-point centered scale, from −3 to +3, indicating markedly worse to markedly improved symptoms. The IC/BPS symptoms were also evaluated using the OSS, including the interstitial cystitis symptom index (ICSI) and interstitial cystitis problem index (ICPI) [45]. Pain severity was assessed using an NRS from 0 to 10 points, with 0 indicating no bladder or pelvic pain and 10 indicating the worst imaginable bladder or pelvic pain [46]. Patients with an NRS over 5 points were classified as bladder pain-predominant subtype NHIC. Satisfactory response was defined as a GRA of moderate (+2) or marked improvement (+3) after treatment. In contrast, treatment outcomes were deemed unsatisfactory if patients did not achieve this level of improvement.

### 5.3. Collection, Measurement, and Analysis of Inflammatory Cytokines and Oxidative Stress Biomarkers in Urine Biomarker Specimens

Fifty milliliters of self-voided urine samples were collected at baseline to analyze urinary biomarkers in all participants. The subjects self-voided when their bladders were full. A urinalysis was performed prior to storage of the samples to ensure there were no infections. The urine was immediately placed on ice and transported to the laboratory for preparation. A 10-minute centrifuge was performed at 4 °C at 1800 rpm; 1.5 mL tubes were used to store 1 mL aliquots of the supernatants at −80 °C. Before further analysis, the urine samples were centrifuged at 12,000 rpm for 20 min at 4 °C. The resulting supernatants were used for subsequent measurements. The urine samples were analyzed for 12 targeted analytes using the Milliplex^®^ Human cytokine/chemokine magnetic bead-based panel kit (Millipore, Darmstadt, Germany). The urinary biomarkers targeted analytes including eotaxin, IL-2, IL-6, IL-8, CXCL10, MCP-1, MIP-1β, RANTES, TNF-α, NGF, BDNF, and PGE2. Additionally, the quantifications of 8-OHdG, 8-isoprostane, and total antioxidant capacity (TAC) were performed on the urine samples. The detailed procedures were similar to those reported in a previous study [47].

### 5.4. Videourodynamic Study Examination

VUDS was performed using a multichannel urodynamic system (Life-Tech, Stafford, TX, USA) and a C-arm fluoroscope (Toshiba, Tokyo, Japan) before the BoNT-A injection. The parameters included the first sensation of bladder filling, the sensation of a full bladder, cystometric bladder capacity, and detrusor pressure at the maximum flow rate. In addition to the IC/BPS diagnosis, lower urinary tract dysfunction was diagnosed based on the VUDS findings as recommended by the International Continence Society [48]. When an intense urge to void or the perception of bladder pain occurred during potassium chloride (KCl) instillation after VUDS, a positive KCl test was considered [49]. Changes in objective parameters were measured by uroflowmetry at baseline and six months after treatment, including maximum flow rate, voided volume, and postvoid residual. All patients were treated with consecutive bladder-targeting medications after cystoscopic hydrodistention, including nonsteroidal anti-inflammatory drugs, cyclooxygenase-2 inhibitors, antimuscarinics, alpha-blockers, intravesical hyaluronic acid instillation, and intravesical BoNT-A injections, as recommended in the AUA and Japanese Urological Association (JUA) guidelines [2,5].

### 5.5. Procedure of Intravesical Botulinum Toxin A Injection

The BoNT-A solution (Allergan, Irvine, CA, USA) was prepared by dissolving 100 U of onabotulinumtoxin A in 10 mL of 0.9% saline. BoNT-A was injected into the bladder wall suburothelially. Twenty injections of BoNT-A liquid, each containing 5 units, were administered in this procedure. A 23-gauge needle and a rigid cystoscopic injection instrument (22 Fr, Richard Wolf, Knittlingen, Germany) were used for injecting at twenty sites along the posterior and lateral walls of the bladder. We injected BoNT-A at the bladder volume around 200 mL, and the injection needle was inserted less than 1 mm into the urothelium, avoiding the trigone [50]. Cystoscopic hydrodistention was performed for 15 min following BoNT-A injections with slowly dripping normal saline at a maximal intravesical pressure of 80 cm H_2_O. The MBC and glomerular grade were determined after the release of intravesical pressure [5]. Hunner lesions and grades of glomerulations were recorded as previously reported. After BoNT-A treatment, a 14-F urinary catheter was inserted overnight and removed the following day. Patients were given a standard antibiotic regimen (Cephradine 500 mg every 6 h) for one week. The outcome assessment was performed at the outpatient clinic.

### 5.6. Statistical Analysis

Statistical analysis was conducted using SPSS version 25 (IBM, Armonk, NY, USA), with statistical significance set at *p*-value < 0.05. Continuous variables are expressed as means and standard deviations, and categorical variables are presented as counts and proportions. Categorical variables were compared using Pearson’s chi-square or Fisher’s exact tests. Continuous variables, excluding urinary biomarker outliers, were compared with independent *t*-tests. First, we divided patients into two subgroups, satisfactory and unsatisfactory groups, and used the t-test to compare the two groups’ subjective and objective parameters. Further, to deeply discover the key to an effective treatment outcome with intravesical BoNT-a in NHIC, a multivariate logistic regression model, which incorporated both subjective and objective factors, including MBC, glomerulation grade, comorbidities, and a bladder pain-predominant or awake urination condition, was used to predict the factors influencing expected treatment outcomes, trying to build a scoring system that expected satisfactory BoNT-A treatment outcomes.

## Figures and Tables

**Table 1:** Baseline characteristics in subjective parameters of patients with non-Hunner IC/BPS according to the treatment outcome (*n* = 220).

Variable		Total (*n* = 220)	Unsatisfactory Outcome GRA ≤ 1 (*n* = 124)	Satisfactory Outcome GRA ≥ 2 (*n* = 96)	*p*-Value
Age (years)		53.64 ± 13.19	52.88 ± 14.35	54.63 ± 11.52	0.318
Gender	Male	42 (19.1%)	26 (19.7%)	16 (16.7%)	0.349
	Female	178 (80.9%)	98 (80.3%)	80 (83.3%)	
IC duration		13.51 ± 10.02	14.07 ± 10.63	12.80 ± 9.19	0.356
Comorbidities		2.83 ± 2.25	2.88 ± 2.22	2.77 ± 2.08	0.713
Numerical rating pain scale		4.93 ± 2.65	4.55 ± 2.73	5.44 ± 2.47	0.004 *
IC symptoms index		12.51 ± 3.74	12.06 ± 3.67	13.10 ± 3.76	0.043 *
IC problem index		11.91 ± 3.32	11.30 ± 3.17	12.71 ± 3.37	0.002 *
Bladder pain-predominant phenotype		126 (57.3%)	58 (46.8%)	68 (70.8%)	<0.001 *

* Significant *p* < 0.05.

**Table 2:** Baseline characteristics in objective parameters of patients with non-Hunner IC/BPS according to the treatment outcome (*n* = 220).

Variable		Total (*n* = 220)	Unsatisfactory Outcome GRA ≤ 1 (*n* = 124)	Satisfactory Outcome GRA ≥ 2 (*n* = 96)	*p*-Value
FSF (mL)		120.32 ± 51.88	120.82 ± 51.39	119.67 ± 52.78	0.871
FS (mL)		186.25 ± 71.96	186.99 ± 73.92	185.29 ± 69.74	0.863
CBC (mL)		284.85 ± 110.12	278.17 ± 102.70	293.47 ± 110.01	0.308
P_det_Q_max_ (cm H_2_O)		21.20 ± 12.88	21.02 ± 12.79	21.43 ± 13.07	0.821
Q_max_ (mL/s)		12.50 ± 5.82	12.15 ± 5.69	12.95 ± 5.98	0.318
Voided volume (mL)		258.93 ± 119.13	248.8 ± 113.17	272.02 ± 125.81	0.152
PVR (mL)		26.44 ± 51.64	28.31 ± 53.91	22.99 ± 48.86	0.426
KCl test—Pain (%)		170 (77.3%)	92 (75.4%)	74 (78.7%)	0.342
KCl test—Urge (%)		66 (30%)	41 (33.6%)	25 (26.6%)	0.169
MBC (mL)		672.59 ± 188.82	647.50 ± 189.21	705 ± 184.26	0.025 *
Glomerulation grade	Grade 1	93 (42.3%)	48 (38.7%)	45 (46.9%)	0.420
	Grade 2	97 (44.1%)	59 (47.6%)	38 (39.6%)	
	Grade 3	30 (13.6%)	17 (13.7%)	13 (13.5%)	

FSF, first sensation of filling; FS, full sensation; CBC, cystometric bladder capacity; P_det_Q_max_, detrusor pressure; Q_max_, maximum flow rate; PVR, postvoid residual; MBC, maximum bladder capacity; * Significant *p* < 0.05.

**Table 3:** Baseline urine biomarker parameters of patients with non-Hunner IC/BPS according to treatment outcome and comparison with control group (NHIC *n* = 220, control = 31).

Variable	Total (*n* = 220)	Unsatisfactory Outcome GRA ≤ 1 (*n* = 124)	Satisfactory Outcome GRA ≥ 2 (*n* = 96)	*p*-Value	Control (*n* = 31)
**Urine Biomarkers**					
IL-8	25.58 ± 74.36	21.63 ± 44.63	31.02 ± 102.32	0.495	12.44 ± 20.97
CXCL 10	17.59 ± 45.04	20.41 ± 49.95	13.71 ± 37.39	0.421	13.81 ± 18.42
MCP-1	316.37 ± 447.38	310.68 ± 492.59	324.18 ± 381.32	0.871	147.13 ± 109.73 *
NGF	0.17 ± 0.0692	0.18 ± 0.08	0.17 ± 0.03	0.557	0.26 ± 0.07 *
BDNF	1.56 ± 9.80	2.28 ± 12.87	0.59 ± 0.16	0.351	0.54 ± 0.11
Eotaxin	6.97 ± 7.47	6.49 ± 6.20	7.62 ± 8.95	0.413	4.97 ± 3.7 *
IL-2	0.21 ± 0.1823	0.22 ± 0.23	0.2071 ± 0.07	0.560	0.80 ± 0.18 *
IL-6	9.09 ± 55.91	10.98 ± 71.45	6.50 ± 21.14	0.666	1.29 ± 1.35
MIP-1β	1.71 ± 4.0471	1.35 ± 2.48	2.19 ± 5.51	0.283	2.52 ± 1.81
RANTES	11.14 ± 64.04	16.82 ± 87.06	5.43 ± 7.35	0.405	6.04 ± 5.15
TNF-α	7.46 ± 37.26	11.23 ± 48.64	2.27 ± 4.48	0.695	0.81 ± 0.32
PGE2	319.72 ± 349.93	346.27 ± 336.51	283.27 ± 367.80	0.130	161.37 ± 105.15 *
8-OHdG	34.68 ± 27.05	36.48 ± 30.10	32.05 ± 22.21	0.375	18 ± 13.73 *
8-Isoprostane	88.77 ± 202.29	120.47 ± 260.04	45.87 ± 46.48	0.022 *	16.78 ± 11.74 *
TAC	1171.75 ± 1175.36	1061.15 ± 1096.02	1314.88 ± 1267.41	0.249	1077.91 ± 925

IL-8, interleukin-8; CXCL10, C-X-C motif chemokine ligand 10; MCP-1, monocyte chemoattractant protein-1; NGF, nerve growth factor; BDNF, brain-derived neurotrophic factor; IL-2, interleukin 2; IL-6, interleukin 6; MIP-1β, macrophage inflammatory protein-1beta; RANTES, regulated upon activation/normal T cell expressed and secreted; TNF-α, tumor necrosis factor-alpha; PGE2, prostaglandin E2; 8-OHdG, 8-hydroxy-2-deoxyguanosine; TAC, total antioxidant capacity; * Significant *p* < 0.05.

**Table 4:** Changes of subjective and objective parameters of patients with non-Hunner IC/BPS according to the treatment outcome (*n* = 220).

Variable	Total (*n* = 220)	Unsatisfactory Outcome GRA ≤ 1 (*n* = 124)	Satisfactory Outcome GRA ≥ 2 (*n* = 96)	*p*-Value
Δ ICSI	−4.14 ± 4.72	−3.12 ± 4.38	−5.46 ± 4.84	0.001 *
Δ ICPI	−3.81 ± 4.93	−3.32 ± 4.57	−6.83 ± 5.06	<0.001 *
Δ NRS	−1.87 ± 3.20	−1.09 ± 2.98	−2.89 ± 3.21	<0.001 *
Δ Qmax	3.03 ± 7.97	2.95 ± 7.60	3.15 ± 8.47	0.858
Δ Voided volume	−26.84 ± 148.24	−19.38 ± 138.82	−36.48 ± 159.87	0.415
Δ PVR	26.44 ± 51.64	21.10 ± 73.55	12.10 ± 58.06	0.343

Δ, changes of variables between baseline and 6 months after treatment. ICSI, interstitial cystitis symptom index; ICPI, interstitial cystitis problem index; NRS, numerical rating scale; OSS, O’Leary–Sant Symptom Score; PVR, postvoid residual; VAS, visual analog scale; * Significant *p* < 0.05.

## Data Availability

Data are available upon request from the corresponding authors.
